# Piezo-type mechanosensitive ion channel component 1: a mechano-bioenergetic transducer in the tumour microenvironment

**DOI:** 10.1080/07853890.2025.2603022

**Published:** 2025-12-24

**Authors:** Yingying Zhang, Chan Gao, Yixuan Li, Qinjiao Fu, Yanzhu Liu, Nan Mo, Juanqing Yue, Ying Wang

**Affiliations:** ^a^ The Fourth School of Clinical Medicine, Zhejiang Chinese Medical University, Hangzhou First People’s Hospital, Hangzhou, Zhejiang, China; ^b^ Hangzhou Normal University, Hangzhou, Zhejiang, China; ^c^ Affiliated Hangzhou First People’s Hospital, School of Medicine, Westlake University, Hangzhou, Zhejiang, China

**Keywords:** Piezo1, TME, mechanical signals, tumour progression, immunotherapy

## Abstract

**Background/objectives:**

As a pivotal mechanosensitive ion channel, Piezo-type mechanosensitive ion channel component 1 (Piezo1) converts mechanical stimuli into biochemical signals that regulate key oncogenic processes, including tumour cell proliferation, migration and invasion. Emerging evidence demonstrates that Piezo1 is widely expressed across various cellular compartments of the tumour microenvironment (TME), and its elevated expression strongly correlates with adverse clinical outcomes. A comprehensive understanding of the complex interactions between Piezo1 activation and cytokine networks in different TME cell populations is therefore essential for developing innovative and effective anti-tumour therapeutic strategies. In this review, we aimed to highlight the molecular mechanisms of Piezo1, systematically elucidating how the mechanical stimulation-Piezo1 signalling pathway within the TME contributes to tumour immune escape and malignant progression. Furthermore, we summarized current research advances in Piezo1-targeting drugs and clinical trials, and discuss strategies to improve tissue specificity while minimizing off-target effects.

**Discussion:**

A comprehensive literature review was conducted, focusing on the specific mechanisms through which Piezo1 regulates endothelial cells, immune cells, cancer-associated fibroblasts and the extracellular matrix within the TME. Activation of Piezo1 in endothelial and immune cells promotes tumour angiogenesis and immune evasion.

**Conclusion:**

Piezo1 plays a critical role in tumour progression and represents a promising therapeutic target for cancer treatment

## KEY MESSAGES

- Piezo1 serves as a central mechanosensitive ion channel that integrates physical and biochemical cues within the tumour microenvironment, driving angiogenesis, immune evasion and extracellular matrix remodelling.
- Cell type-specific regulation of Piezo1 in endothelial cells, immune cells and cancer-associated fibroblasts establishes a complex ‘mechano-immuno’ network that critically shapes tumour progression.
- Targeting Piezo1 and its downstream mechanotransduction pathways represents a promising yet challenging therapeutic strategy, requiring precise modulation of its signalling specificity and tissue selectivity.

## 1. Introduction

With the growing understanding of cancer development mechanisms, it is now evident that tumour growth and metastasis are closely associated with the surrounding environment [1]. The tumour microenvironment (TME), the internal milieu where tumour cells originate and reside, comprises various cellular and non-cellular components, including blood vessels, immune cells, fibroblasts, various signalling molecules, and the extracellular matrix (ECM). These components were once considered passive bystanders in tumourigenesis [2]. However, mechanistic studies have revealed that the TME is a highly organized ecosystem in which interactions among cells and molecules form complex signalling networks that promote angiogenesis, induce immune tolerance [3], and actively drive cancer progression. In solid tumours, uncontrolled cell proliferation within a confined space, combined with high vascular permeability, inadequate lymphatic drainage and excessive deposition of ECM proteins, gives the TME a characteristic high stiffness [4]. Physical factors were largely overlooked in earlier studies [5]. However, recent research has demonstrated that mechanical signals can significantly influence cellular behaviour and play key roles in cancer initiation, recurrence and metastasis [6–8]. This recognition has sparked growing interest in mechanosensitive ion channels (MIChs), such as the Piezo-type mechanosensitive ion channel component (Piezo) family, which are widely distributed in animal and human cells and tissues and play vital roles in numerous physiological and pathological processes (Table 1).

**Table 1.** Classification of mechanosensitive ion channels and their pathophysiological functions.

Classification	Functions	Refs
MscL	Avoid bacterial cell cracking in hypotonic environment	[108]
MscS	Involved in the regulation of osmotic pressure to help bacteria adapt to different growth environments	[109]
MSL	MSLs in group I and group II are expressed in mitochondria and plastids, where they have an osmoregulatory role. Group III MSLs reside at the plasma membrane, where their roles remain an active area of research	[85,110]
K2P		
TREK-1	It plays a key role in neural protection, anesthesia, pain perception, and depression at the cellular level, and may provide potential therapeutic targets for liver fibrosis, pancreatic cancer, and others	[111,112]
TREK-2	It plays an important role in physiological and pathological states such as cerebral ischemia, neuroprotection, and memory impairment	[113]
TRAAK	It plays a role in thermally abnormal pain, neurodevelopmental disorders, and the maintenance of resting membrane potential and the conduction of fast action potentials	[114,115]
Piezos		
Piezo1	It is widely expressed in tissues and organs, not only involved in vascular development, blood pressure regulation, cell migration, but also plays an important role in the formation and development of various types of cancer	[95,116]
Piezo2	It is mainly expressed in primary sensory neurons and is related to tenderness, airway extension and proprioception	[117]
OSCA	It plays a role in osmotic induction and stomatal immunity of plants	[118,119]
TMEM63A/B	Control the release of pulmonary surfactant	[120]
TRP		
NOMPC	It plays an important role in balance, tactile and auditory perception mechanisms in nematodes, zebrafish and Drosophila melanogaster	[121]
TRPV4	Participate in vascular function, skin barrier function, joint function, airway function, inner ear function and pain and other aspects	[122]
MET	It is beneficial for cochlear hair cells to convert the mechanical vibration generated by sound waves into electrical signals, thus sensing sound	[123]
DEG	Sense mechanical stimulation, participate in nerve conduction and maintain the balance of salt and water in the body	[124]
ENaCs	It not only participates in the reabsorption of sodium ions but also interacts with neuropeptides	[124,125]
ASICs	Widely distributed around and in the central nervous system, it plays a key role in the onset and progression of various neurological disorder	[126]

mechanosensitive channel large conductance (MscL); mechanosensitive channel small conductance (MscS); MscS-like (MSL); two-pore potassium channel (K2P); hyperosmolality-gated calcium-permeable channels (OSCA); transmembrane protein 63 (TMEM63); transient receptor potential (TRP) channels; no mechanoreceptor potential C (NOMPC); transient receptor potential vanilloid-4 (TRPV4); mechano-electrical transduction (MET) channel complex; degenerin (DEG); epithelial Na + channels (ENaCs); acid-sensing ion channels (ASICs).

In 2010, Ardem Patapoutian ‘s laboratory first identified the Piezo family of mechanosensitive cation channels in mammals. These channels open in response to mechanical stimulation, allowing positively charged ions (including calcium) to enter the cell and activate a cascade of downstream signalling pathways. The Piezo family includes two members: Piezo1 and Piezo2 [9]. Piezo2 primarily mediates tactile, proprioceptive and visceral mechanical stimuli [10], whereas Piezo1 is broadly expressed and regulates essential cellular processes such as proliferation, differentiation, migration and apoptosis [11–13]. Piezo1 also plays crucial roles in various pathological processes, including the modulation of inflammatory responses through cytokine release [14] and the promotion of cancer progression by facilitating tumour cell invasion, metastasis and angiogenesis [15].

When normal tissue transforms into solid tumour tissue, its mechanical stiffness typically increases. This elevated stiffness can further activate signalling pathways related to tumour cell proliferation and invasion by stimulating the mechanosensor Piezo1, promoting cancer cell proliferation, migration, invasion, angiogenesis and immune evasion [4,5]. The increase in ECM stiffness represents more than a physical alteration.

Within stiff matrices, collagen fibres shift from a disordered configuration to ‘dense, parallel bundles’ with enhanced cross-linking [16]. This structural reorganization exposes additional cryptic degradation sites within ECM molecules, facilitating their accessibility to degradative enzymes. Furthermore, stiffness-induced activation of Piezo1 significantly upregulates the expression of these enzymes. Together, these two mechanisms synergistically accelerate cancer cell invasion [17,18]. In breast cancer [19], tumour cells experience significant compressive stress, which activates the Piezo1 channel. The resulting calcium influx triggers several downstream signalling pathways, including Src and extracellular signal-regulated kinase (ERK), leading to ECM protein degradation and thereby enhancing the invasive capacity of cancer cells.

**Scheme 1.**
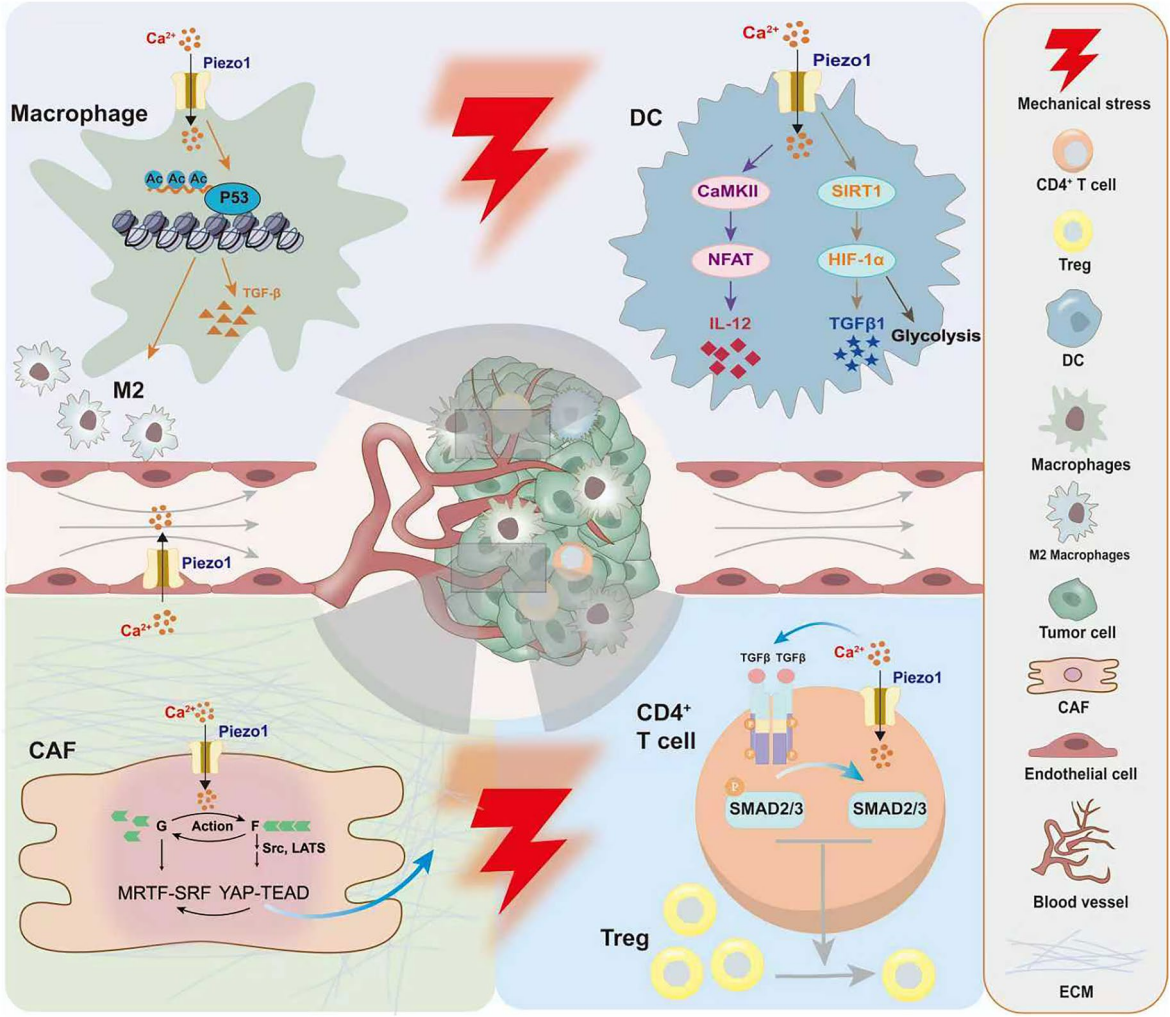
Regulation of the tumour microenvironment by Piezo1.

In recent years, growing evidence has shown that the cellular composition and functional states of the TME also play critical roles in cancer progression. Interestingly, studies have demonstrated that many cells within the TME, such as endothelial cells [20], immune cells [21] and fibroblasts [22], express Piezo1. Piezo1 regulates interactions among various components of the TME and contributes to disease progression. This biological process may represent a promising target for anti-tumour therapy. In this review, we aimed to discuss how aberrant Piezo1 expression in TME cells regulates intercellular and cell-matrix communication and how it influences angiogenesis, immune evasion and ECM remodelling (Scheme 1).

## 2. Structure and function of Piezo1

**Figure 1.**
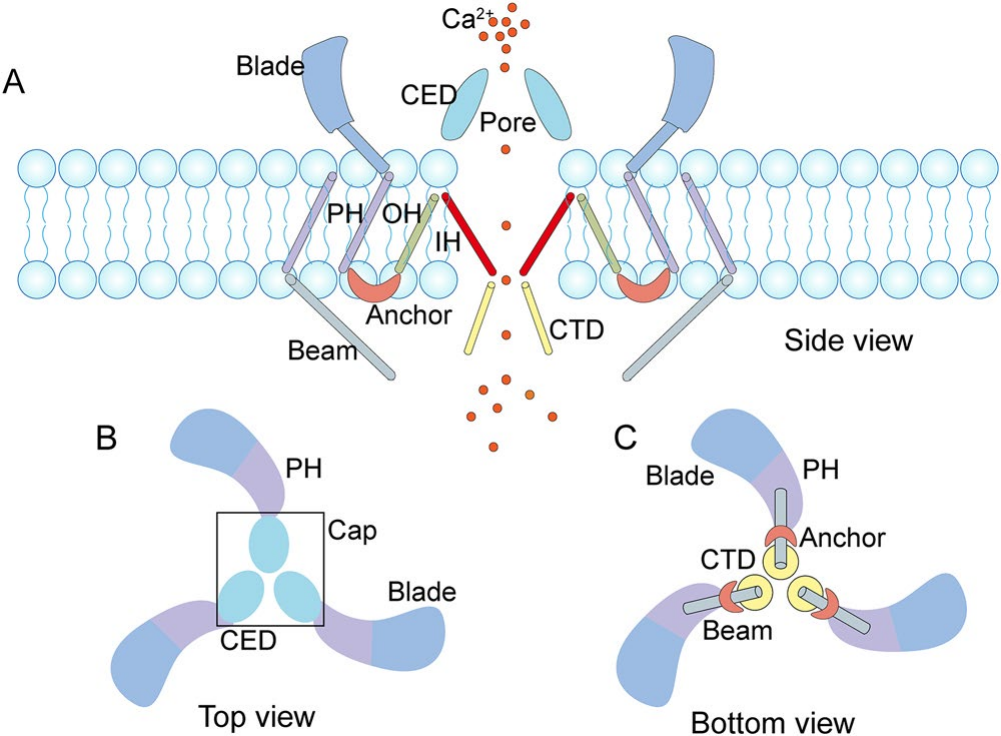
Structure of Piezo1 Channel. (A) Side view of the Piezo1 ion channel. (B) Top view of the Piezo1 ion channel. (C) Bottom view of the Piezo1 ion channel. the major elements composing the ion channel are shown. CED: C-terminal extracellular domain; CTD: intracellular C-terminal domain; OH: outer helix; IH: inner helix; PH: peripheral helix.

Piezo1 is a trimeric, propeller-shaped mechanosensitive channel protein containing 2,547 amino acids and has a molecular weight of approximately 286 kDa (Figure 1). Under electron microscopy, the Piezo1 channel exhibits a central and a peripheral region. The central region forms the cation-conducting pore and includes the inner helix (IH), C-terminal extracellular domain (CED), outer helix (OH) and intracellular C-terminal domain (CTD) [23].

The IH and OH constitute the core transmembrane segments, which, together with the CED and CTD, form the ion permeation pathway that confers ion conductivity and selectivity to Piezo1 [24]. The peripheral region consists of a central anchor, three long beams and three blade-like structures. The anchor contains four consecutive α-helices located at the interface between adjacent subunits, forming a unique hairpin structure roughly parallel to the membrane surface, in which likely stabilizes the trimeric conformation [25,26]. The long beams interact with the blade region of the CTD and the anchor domain, amplifying the mechanical force applied to the blades through a lever-like mechanism. When external mechanical stimuli occur [27], the blades detect membrane deformation, causing the beams to pull on the central pore and open the pore cap, thereby allowing calcium ions (Ca^2+^) to flow through (Figure 1). Using single-molecule fluorescence imaging, researchers have shown that each Piezo1 blade comprises nine repetitive domains. The distal ends of the blades are highly curved and mobile, and their conformational changes are associated with both chemical and mechanical regulation of channel activation. When the Piezo1 channel inhibitor GsMTx-4 is applied, the blades of Piezo1 become compressed, suppressing channel activity. Conversely, osmotic swelling-induced plasma membrane expansion can directly activate the Piezo1 channel. Interestingly, the small-molecule agonist Yoda1 induces strong Ca^2+^-dependent Piezo1 activation similar to that observed with osmotic swelling [28]. Together, these findings provide valuable insight into how Piezo1 is activated and inhibited within the cellular environment.

As a mechanoreceptor, the unique structure of Piezo1 enables it to respond to both external mechanical stimuli and internal cellular signals, converting mechanical forces into electrochemical signals that activate a series of downstream pathways. This process not only regulates specific pathophysiological responses but also influences distinct cellular behaviours. Studies have shown that a stiffer mechanical microenvironment significantly induces overexpression of Piezo1 in human dermal fibroblasts (HDFs) [22,29]. This process is initiated when increased substrate stiffness promotes conformational changes and clustering of integrins on the HDF surface. These activated integrins recruit intracellular focal adhesion proteins to form stable integrin-focal adhesion complexes, which connect to the actin-myosin cytoskeleton, transmitting the stiffness signal into the cell. A particularly rigid microenvironment further activates myosin light-chain kinase (MLCK), inducing actin cytoskeleton contraction and generating ‘mechanical tension’. This tension directly acts on Piezo1 channels on the plasma membrane, shifting them into a ‘preactivated’ state. Preactivated Piezo1 then initiates intracellular signalling cascades that convert the mechanical signal into a ‘transcriptional regulatory signal’ *via* kinase-mediated pathways, ultimately driving Piezo1 gene overexpression [4,30]. Elevated Piezo1 overexpression enhances the sensitivity of HDFs to mechanical stimuli, making the cells more responsive to changes in stiffness. The subsequent Ca^2+^ influx further activates downstream pathways, contributing to hypertrophic scar formation [22]. The precise mechanisms underlying this phenomenon remain unclear; however, they may involve the transforming growth factor-beta 1 **(**TGF-β1)/Smad and Yes-associated protein (YAP)/transcriptional coactivator with PDZ-binding motif (TAZ) signalling pathways [31,32]. Importantly, Piezo1 is highly expressed in various tumours. Numerous studies have demonstrated that Piezo1 plays a critical role in the mechanically induced proliferation and migration of prostate [33], ovarian [34] and gastric cancer [35] cells. Therefore, this mechanosensitive signalling mechanism may represent a promising therapeutic target for tumour therapy.

## 3. Composition of the tumour microenvironment

The TME comprises complex and diverse components, including endothelial cells, various immune cells, tumour-associated fibroblasts and the ECM [1], which collectively surround cancer cells and contribute to the dynamic, vascularized progression of malignant tumours [36].

### 3.1. Vascular cells

Vascular cells primarily include vascular endothelial cells, lymphatic endothelial cells and pericytes, among which vascular endothelial cells are of greatest interest. In the TME, Piezo1 functions as a mechanosensor in vascular endothelial cells. By detecting mechanical stimuli such as blood flow shear stress and ECM stiffness, it regulates vascular development and neovascularization [5]. The Piezo1 channel rapidly inactivates in heterologous expression systems; however, its behaviour is altered within the TME. The combination of dynamic mechanical fluctuations from constant ECM remodelling, cytoskeletal alterations, a stiffness-induced ‘pre-activated’ state, and the engagement of non-inactivating gating modes collectively convert Piezo1 into a chronic mechanical sensor [37,38]. Consequently, the elevated interstitial pressure prevalent in tumour tissues persistently activates Piezo1, leading to pathological angiogenesis characterized by vessel tortuosity and leakage, which in turn facilitates tumour cell invasion and migration [5,39]. Concurrently, local tissue hypoxia or inflammation promotes new blood vessel formation. Piezo1 detects mechanical stimulation and activates actin reorganization *via* the Ca^2+_^ RhoA/ROCK pathway (Table 2), thereby promoting endothelial cell migration towards hypoxic areas [32,39]. These findings indicate that endothelial cell-expressed Piezo1 contributes to the malignant progression of tumours, which will be elaborated upon later.

**Table 2.** List of abbreviations.

Abbreviation	Full term in English
Arg-1	arginase-1
CAFs	cancer-associated fibroblasts
CCL2	C C motif ligand 2
CED	C-terminal extracellular domain
CTD	intracellular C-terminal domain
CTGF	connective tissue growth factor
CXCL12	C-X-C motif chemokine ligand 12
CXCL16	C-X-C motif chemokine ligand 16
DCs	Dendritic cells
ECM	the extracellular matrix
ECs	endothelial cells
ERK	extracellular signal-regulated kinase
FAK	Focal adhesion kinases
HCC	hepatocellular carcinoma
HDFs	human dermal fibroblasts
HIF-1α	hypoxia-inducible factor 1α
HUVECs	human umbilical vein endothelial cells
IFNγ	Interferon gamma
IGFBP2	Insulin-like Growth Factor Binding Protein 2
IH	inner helix
IL	Interleukin
IS	immunological synapse
K2P	two-pore potassium
LPS	Lipopolysaccharide
MCP-1	Monocyte Chemoattractant Protein-1
MDSCs	Myeloid-derived suppressor cells
MIChs	Mechanosensitive ion channels
MLC	Myosin Light Chain
MLCK	myosin light-chain kinase
MMP-2	matrix metalloproteinase-2
MMPs	Matrix metalloproteinases
MRTF	myocardin related transcription factor A
MT1-MMP	membrane-type matrix metalloproteinase-1
NETosis	neutrophil extracellular trap formation
NFAT	Nuclear Factors of activated T cells
NF-κB	Nuclear factor kappa-B
OH	outer helix
OSCA	hyperosmolality-gated calcium-permeable channels
Osr2	Odd-skipped related transcription factor 2
PD-1	programmed cell death protein 1
PPARγ	peroxisome proliferator activated receptorγ
PYK2	Proline-rich tyrosine kinase 2
RhoA	Ras Homolog Family Member A
ROCK	Rho-associated kinase
ROS	Reactive oxygen species
S1P	sphingosine-1-phosphate
SDHA	Succinate dehydrogenase complex flavoprotein subunit A
SIRT1	sirtuin 1
SIRT3	sirtuin 3
Src	Steroid receptor coactivator
SRF	Serum response factor
STAT6	Signal Transducer And Activator Of Transcription 6
TAECs	Tumour-Associated Endothelial Cells
TAMs	tumour-associated macrophages
TANs	tumour-associated neutrophils
TAZ	Transcriptional coactivator with PDZ-binding motif
TGF-β1	transforming growth factor-β1
TME	the tumour microenvironment
TMEM63	transmembrane protein 63
TNF-α,	Tumour Necrosis Factor α
Tregs	Regulatory T cells
TREK-1	TWIK-Related K+ Channel 1
TRP	transient receptor potential
VEGF	vascular endothelial growth factor
WSS	wall shear stress
YAP	Yes-associated protein
α-SMA	Alpha smooth muscle actin

### 3.2. Cancer-associated fibroblasts and the extracellular matrix

Cancer-associated fibroblasts (CAFs) are a crucial component of the tumour stroma. In recent years, the significant plasticity of CAFs has garnered increasing interest [40]. During the regulation of inflammatory factor secretion, the high stiffness of tumour tissue activates the Piezo1-Ca^2+^ axis in CAFs. The synergistic effect of this Ca^2+^ influx and the reinforcement of cytoskeletal tension disrupts the cytoplasmic sequestration of YAP/TAZ, enabling their sustained nuclear translocation and transcriptional activity. Within the nucleus, YAP/TAZ form complexes with TEAD family transcription factors, which directly bind to the promoters of genes encoding interleukin (IL)-6, IL-8 and monocyte chemoattractant protein-1 (MCP-1), thereby promoting the secretion of these factors. This cascade fosters an immunosuppressive microenvironment that facilitates malignant progression [41,42]. Furthermore, within the TME, Piezo1-YAP/TAZ-driven cytokine secretion is not an isolated event. Inflammatory factors such as IL-6 and tumour growth factor (TGF)-β1 can, in turn, activate CAFs, enhancing collagen deposition and cross-linking in the ECM, which further increases matrix stiffness. This process creates a vicious cycle between the aberrant mechanical microenvironment and tumour progression, continuously fuelling malignancy [14,43].

The ECM is the non-cellular structural component of the TME, composed of fibrous proteins such as collagen, glycoproteins and proteoglycans that mediate intercellular communication [1]. Piezo1 regulates ECM remodelling to promote tumour cell invasion. The Piezo1-Ca^2+^ axis facilitates tumour invasion either by increasing matrix metalloproteinase (MMP) secretion to degrade the basement membrane or by inducing CAFs to produce aligned collagen fibres [27]. Notably, upon Piezo1 activation, CAFs become infiltrative and highly activated, upregulating the synthesis of lysyl oxidase and collagen, thereby promoting ECM fibrosis and stiffening. The resulting increase in ECM stiffness further activates Piezo1 in tumour cells, creating a vicious cycle [5,16]. Disrupting this cycle may therefore represent a potential direction for cancer therapy.

### 3.3. Immune cells

Immune cells are categorized into innate and adaptive immune cells, which perform essential roles in immune defence, surveillance and homeostasis, serving as the body’s protective guardians. However, under certain pathological conditions, immune cells may become dysfunctional or act as aggressors, thereby exacerbating disease progression.

Within the TME, Piezo1 promotes immune evasion and malignant progression by regulating the mechanical sensing, polarization, and functional states of immune cells. The high stiffness of the tumour ECM activates Piezo1 in macrophages, triggering the STAT6/PPARγ signalling pathway, which promotes M2 polarization and suppresses anti-tumour immunity [44] (Table 2). Myeloid-derived suppressor cells (MDSCs) inhibit T cell function, and Piezo1 can promote MDSC expansion and recruitment by inhibiting retinoblastoma 1 [45]. In tumour-associated neutrophils (TANs), Piezo1 activation induces Ca^2+^-dependent neutrophil extracellular trap formation (NETosis), releasing deoxyribonucleic acid networks that capture circulating tumour cells and facilitate their metastasis and colonization [46]. Moreover, the high-stiffness ECM activates the Ca^2+^-NFAT pathway in T cells *via* Piezo1, upregulating immune checkpoint molecules such as programmed cell death protein 1 (PD-1) and T-cell immunoglobulin and mucin-domain containing-3, which drive T-cell exhaustion [47]. Moreover, fibrotic ECM physically impedes T-cell infiltration, enabling tumour immune escape. In recent years, such an immunosuppressive microenvironment has contributed to the limited efficacy of immunotherapy in many cancers [47,48]. Therefore, alleviating immune suppression within the TME has become essential. Targeting the Piezo1 pathway or reducing ECM stiffness may provide promising therapeutic strategies to overcome tumour-induced immune inhibition.

## 4. Regulation of endothelial cell biological activities by Piezo1

Endothelial Cells (ECs), which line the vascular system, form a selective barrier at the blood-tissue interface and play a central role in physiological processes such as angiogenesis, hemodynamic regulation and vascular tone maintenance. As mechanosensitive cells, ECs sense and transduce mechanical signals, including blood flow shear stress and ECM stiffness, primarily through Piezo1, playing a key role in endothelial barrier integrity and vascular homeostasis [49,50]. Within the TME, Tumour-Associated Endothelial Cells (TAECs) exhibit significant pathological characteristics, including structural abnormalities, functional impairment and dysregulated immunomodulatory activity. Piezo1 significantly promotes abnormal tumour angiogenesis and immune evasion by regulating signal transduction in TAECs [35,51].

### 4.1. Angiogenesis

Angiogenesis is regulated by a dynamic balance between pro-angiogenic and inhibitory factors. When pro-angiogenic signalling pathways predominate, endothelial cells become activated. Within the TME, various cytokines secreted by tumour cells abnormally activate TAECs, inducing endothelial sprouting and initiating pathological angiogenesis [52]. Hypoxia is a key pathophysiological driver of angiogenesis and is particularly prominent in the core regions of solid tumours [5]. Under hypoxic conditions, stabilized hypoxia-inducible factor 1α (HIF-1α) transcriptionally activates multiple pro-angiogenic genes, such as vascular endothelial growth factor (VEGF), MMPs and specific integrins, to enhance vascular permeability and promote the extension and stabilization of new blood vessels [53].

Beyond biochemical stimuli, mechanical force signals also play a critical role in angiogenesis [5]. Bioinformatics analyses have revealed that Piezo1 expression is elevated in high-stiffness matrices and positively correlates with CD31 expression. In an established Sprague–Dawley rat model with a high liver stiffness background, Piezo1 knock down reduced CD31 expression and tissue microvessel density, significantly inhibiting lung metastasis. Both bioinformatic analysis and *in vivo* experiments indicated that Piezo1 mediates matrix stiffness-induced angiogenesis in hepatocellular carcinoma (HCC). To further explore this mechanism, conditioned media collected from control or shPiezo1-transfected HCC cells grown on high-stiffness matrices were used to treat human umbilical vein endothelial cells (HUVECs). The control group exhibited stronger tube formation and migration abilities. Using a human angiogenesis array containing 55 cytokines, three differentially expressed pro-angiogenic factors were identified: VEGF, CXCL16 and IGFBP2 (Table 2). These findings suggest that Piezo1 upregulation promotes HCC angiogenesis by modulating the expression and secretion of pro-angiogenic factors. This study further linked HIF-1α, Piezo1 and pro-angiogenic factors, elucidating that high stiffness-induced Piezo1 activation significantly upregulates HIF-1α and its downstream targets, including VEGF, IGFBP2 and CXCL16, thereby promoting HCC angiogenesis. Similarly, during vascular development, wall shear stress (WSS) and the pro-angiogenic mediator sphingosine-1-phosphate (S1P) regulate capillary morphogenesis and branching patterns by activating Piezo1 [51].

When Piezo1 was specifically knocked out in endothelial cells, calcium influx defects and disrupted endothelial cell alignment were observed under WSS and S1P stimulation. Pharmacological inhibition of Piezo1 activity, combined with WSS and S1P stimulation, failed to restore endothelial sprouting and lumen formation. Further experiments revealed that S1P, similar to WSS, activates MMP-2 and membrane-type MMP-1 (MT1-MMP) through Piezo1-Ca^2+^ gating, promoting vascular sprouting. Collectively, these studies [5,51] identify Piezo1 as a central hub integrating mechanical and biochemical signals, mediating calcium-dependent pathways that cooperatively promote angiogenesis.

Notably, preclinical studies have confirmed that inhibiting VEGF signalling effectively suppresses tumour angiogenesis. The convergence of mechanical and biochemical signalling at Piezo1 provides a strong rationale for developing novel anti-angiogenic strategies. This discovery expands the conventional understanding of angiogenesis regulation – previously centred primarily on biochemical factors – and holds translational potential in clinical oncology.

### 4.2. Facilitating immune evasion

In the TME, endothelial cells not only promote tumour angiogenesis and form abnormal vascular structures through Piezo1 activation but also facilitate immune evasion through multiple mechanisms. Studies have shown that sustained Piezo1 activation strengthens the physical barrier, upregulates immune checkpoint molecules, induces metabolic immunosuppression, and disrupts chemokine gradients, thereby inhibiting T cell infiltration and function [38,47,54,55].

Research has demonstrated that Piezo1 plays a crucial role in leukocyte transendothelial migration. In inflammatory models, Piezo1 facilitates endothelial barrier opening *via* the Src/PYK2/MLC pathway (Table 2), promoting leukocyte (including T cell) transendothelial migration [56], exerting normal immunomodulatory functions. However, within the TME, matrix stiffness may induce endothelial cell contraction through Piezo1 activation, increasing vascular permeability to facilitate tumour cell metastasis while simultaneously forming a physical barrier that limits T cell infiltration into tumour tissue [5,27]. The formation of an immunosuppressive microenvironment is closely related to Piezo1 activity. Endothelial cell Piezo1 activation leads to the secretion of immunosuppressive cytokines such as TGF-β and IL-10, inhibiting T cell proliferation and cytotoxic activity [57]. Furthermore, Piezo1 activates nuclear factor kappa B (NF-κB) *via* calcineurin, further upregulating the expression of pro-inflammatory cytokines and immune checkpoint molecules (such as PD-L1) [57–59]. TAECs within the TME secrete chemokines (e.g. CCL2, CXCL12), preferentially recruiting immunosuppressive cells (e.g. Tregs, MDSCs) rather than cytotoxic T cells [5,60]. These results collectively demonstrate that activating Piezo1 not only promotes the formation of abnormal vascular structures that sustain tumour growth but also regulates endothelial cells to promote tumour immune evasion.

Within the complex network of the TME, Piezo1 regulates inflammatory responses, angiogenesis, and immune cell recruitment through endothelial cell mechanosensing, thereby creating a microenvironment that promotes tumour immune evasion. However, its specific regulatory mechanisms require further investigation. Future studies integrating single-cell sequencing and *in vivo* imaging to characterize the dynamic role of Piezo1 in tumour vasculature may provide new avenues for developing Piezo1-targeted immunotherapies.

## 5. Regulation of immune cells in the tumour microenvironment by Piezo1

**Figure 2.**
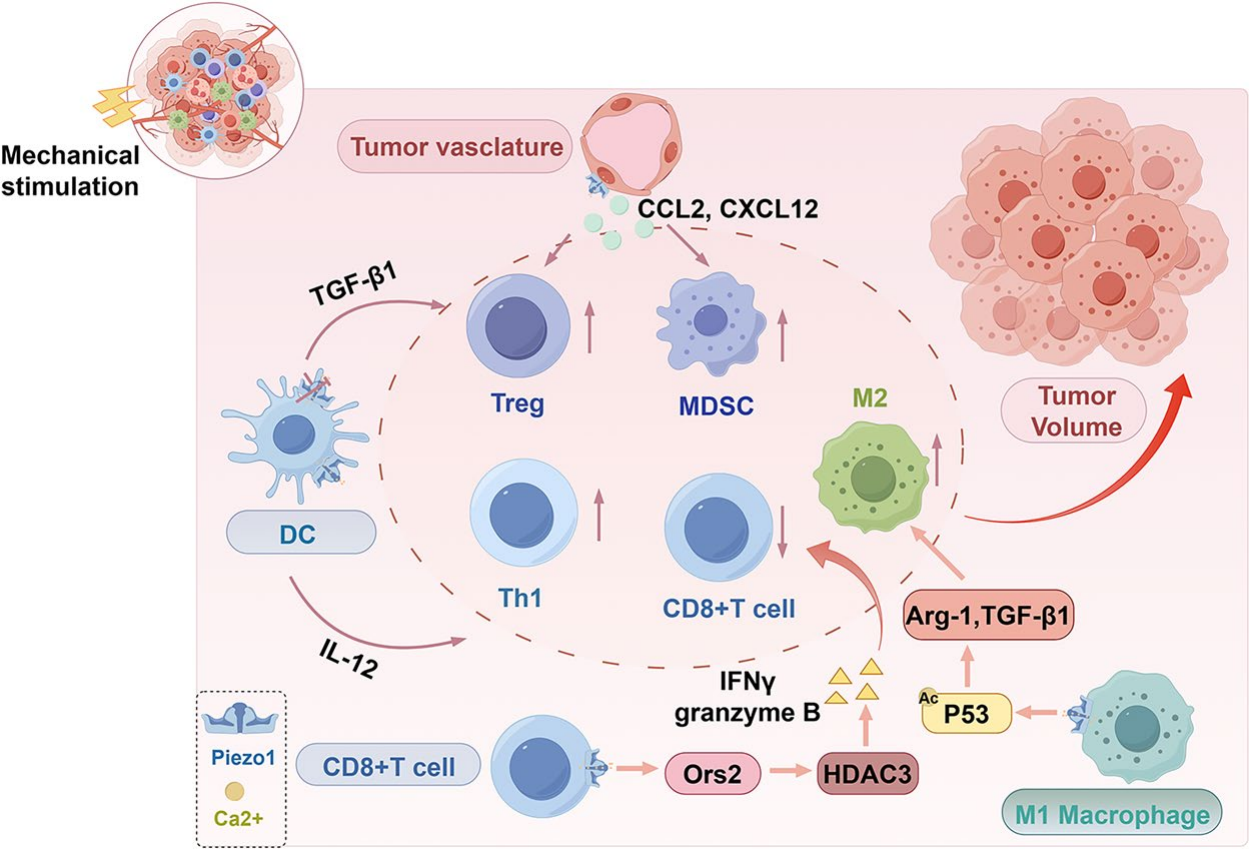
The regulatory effect of mechanical stimulation-activated Piezo1 on immune cells in the tumour microenvironment.

Piezo1, acting as a mechanosensor within the tumour microenvironment, serves as a key node linking mechanical cues to immune responses by regulating immune cell function [45] at multiple levels (Figure 2).

### 5.1. Role of Piezo1 in innate immunity

Within the tumour microenvironment, cytokines drive macrophage differentiation into different types of tumour-associated macrophages (TAMs), primarily classified as M1 and M2. M1 macrophages are generally considered tumouricidal, exhibiting anti-tumour and pro-inflammatory properties, whereas M2 macrophages exhibit immunosuppressive and pro-tumourigenic functions. As the tumour progresses, increasing matrix stiffness activates macrophage Piezo1, promoting polarization of TAMs towards the M2 phenotype and shifting their function from pro-inflammatory to immunosuppressive [61]. Studies have shown that [61,62] stiff matrices activate the Ca^2+^-calmodulin pathway in macrophages *via* Piezo1, inducing p53 deacetylation and subsequently upregulating arginase-1 (Arg-1) and TGF-β1 expression, driving M2 polarization. Piezo1 also regulates macrophage oxidative phosphorylation through the SIRT3-SDHA-HIF-1α axis, enhancing the secretion of anti-inflammatory cytokines such as IL-10 [63] (Table 2). This mechanism likely contributes to the maintenance of the immunosuppressive TAM phenotype. Furthermore, during infection or within the TME, Piezo1 forms a complex with the Toll-like receptor 4, cooperatively activating the NF-κB pathway to enhance macrophage phagocytic capacity and reactive oxygen species (ROS) production [64]. However, this excessive activation may result in uncontrolled inflammation, ultimately facilitating tumour progression.

Dendritic cells (DCs), the key antigen-presenting cells that bridge innate and adaptive immunity, play a central role in anti-tumour immune responses. Recent studies have highlighted the significant role of Piezo1 in regulating DC function. Notably, Piezo1 integrates the SIRT1-HIF-1α-glycometabolism axis with the Ca^2+^-NFAT pathway, thereby coordinating the balance between TGFβ1 and IL-12 secretion [21,65]. This finding establishes Piezo1 as a central hub in multiple signalling networks, functioning not merely as a mechanical stimulus transducer but as a ‘molecular decider’ that directs immune responses by coupling metabolic reprogramming with calcium signalling. Under conditions of increased matrix stiffness or inflammatory stimulation, Piezo1 activation promotes IL-12 secretion, driving Th1 cell differentiation and enhancing anti-tumour immunity. In contrast, Piezo1 deficiency elevates TGFβ1 levels, promoting Treg cell expansion [45,65,66]. This mechanism, which integrates distinct pathways to regulate key cytokines, provides a unified framework for understanding Piezo1’s complex role in immune regulation.

Current evidence supports a model in which Piezo1 functions as an ‘integration platform’ for extracellular and intracellular signals, unifying its complex downstream pathways. Its functional output depends on the relative weighting and crosstalk among different signalling pathways within a given microenvironment. In mechanically rich, stiff environments, Piezo1 preferentially activates a pro-inflammatory axis [67], whereas under specific pathological conditions, the signalling balance may shift towards immunosuppression [38]. This context-dependent integration enables a single molecule to coordinate diverse immune functions and explains the functional heterogeneity of Piezo1 across different immune cell types. Understanding Piezo1 in this framework is crucial for developing novel therapies targeting tumour immune evasion, positioning Piezo1 as a tunable signalling hub rather than a simple mechanosensor and guiding the design of more precise immunomodulatory strategies.

### 5.2. Role of Piezo1 in adaptive immunity

Adaptive immunity encompasses the process by which antigen-specific B and T lymphocytes, upon encountering antigenic stimulation, become activated, proliferate, differentiate into effector cells, and execute their biological functions. Central to this process is the interaction between the T cell receptor and major histocompatibility complex molecules on antigen-presenting cells, which forms a dynamic supramolecular structure at the cell membrane called the immunological synapse (IS). This interaction triggers cytoskeletal rearrangements and activates downstream signalling pathways, enabling effective T cell activation and effector function [68].

Recent studies indicate that T cells are mechanosensitive, with Piezo1 playing a significant role in human T cell activation. During IS formation, mechanical stimulation from membrane stretch activates Piezo1, inducing calcium influx and activating calpain, which drives actin cytoskeleton remodelling and T cell activation. However, within the TME, Piezo1 inhibition enhances the mechanical cytotoxicity of T cells against tumour cells. Even in the absence of cytokine secretion, T cells can transmit mechanical force *via* the IS to induce tumour cell apoptosis [38]. In fibrotic regions of solid tumours, a high-stiffness matrix activates Piezo1 on CD8+ T cells, triggering Ca^2+^ influx and activating the Osr2 transcription factor. Osr2 recruits histone deacetylase 3, suppressing the expression of cytotoxic genes such as IFNγ and granzyme B, leading to terminal T cell exhaustion. For example, samples of patients with liver cancer show that CD8+ T cells in fibrotic regions highly express Osr2 and PD-1. Blocking Piezo1 reduces Osr2 expression and restores T cell proliferation and cytotoxic capacity [47]. Therefore, combining anti-PD-1 antibodies with Piezo1 inhibitors (e.g. GsMTx4) significantly enhances T cell infiltration and tumour suppression [38,47]. Furthermore, some studies indicate that Piezo1 can selectively inhibit Tregs without affecting effector T cell function. Piezo1 knockout in T cells activates the TGF-β/SMAD pathway, enhancing SMAD2/3 phosphorylation and Treg expansion, suggesting that Piezo1 activation in T cells enhances immune responses [69]. As previously discussed regarding DCs, Piezo1 responds to inflammatory and mechanical stimuli by regulating IL-12 and TGF-β1 secretion, modulating T cell surface receptor signalling, and modulating Th1 and Treg differentiation [65]. Collectively, these findings highlight Piezo1 as an indispensable regulator of T cell subset differentiation.

In fact, nearly all progressing tumours induce varying degrees of T cell, macrophage, and DC exclusion or trigger dysfunction programs in CD8+ T cells, creating a microenvironment that supports tumour growth. Therefore, activating and enhancing the anti-tumour functions of immune cells is critically important. However, strategies targeting Piezo1 for tumour immunotherapy face significant challenges. The differential regulation of Piezo1 across immune cell types, combined with the need to selectively modulate Piezo1 in intratumoural T cells without disrupting peripheral immune homeostasis, necessitate in-depth elucidation of molecular mechanisms and optimization of delivery systems for clinical translation.

## 6. Role of Piezo1 on CAFs

CAFs are major components of the TME, remodelling ECM through secretion of soluble molecules and establishing connections with tumour cells and other TME components, thereby promoting tumour growth and invasion [70]. In addition, CAFs play a significant role in regulating tumour immunity [60]. Consequently, increasing attention has focused on the molecular mechanisms by which CAFs promote cancer progression.

Chronic inflammation, a hallmark of the TME, directly targets Piezo1 on CAFs to initiate a highly efficient cytokine secretion program. This process begins when inflammatory mediators such as TNF-α, IL-1β, or LPS activate canonical inflammatory signalling pathways, including NF-κB, through their respective receptors, subsequently upregulating Piezo1 expression in CAFs and enhancing their sensitivity to mechanical stimuli [40,43]. Direct evidence in CAFs is still emerging; however, multiple studies in immune and cancer cells validate this regulatory mechanism. For example, LPS stimulation significantly increases Piezo1 expression in macrophages and astrocytes [71,72], suggesting that a similar mechanism likely operates in CAFs within inflammatory microenvironments. Inflammation primes CAFs to respond more effectively to mechanical cues. Concurrently, inflammation-induced tissue damage and fibrosis increase matrix stiffness, serving as a persistent agonist for Piezo1. This physical signal synergizes with chemical inflammatory cues to robustly activate Piezo1 channels on CAF membranes, triggering substantial Ca^2+^ influx. The incoming Ca^2+^ acts as a critical second messenger that activates two major pathways: it stimulates the YAP and MRTF-SRF regulatory networks to drive transcriptional reprogramming [73,74], and it synergizes with inflammatory signals to amplify NF-κB transcriptional activity. In gastric cancer, this pathway also involves activation of the Piezo1/YAP1/CTGF axis [43]. These simultaneously activated downstream pathways converge in the nucleus to drive extensive transcriptional reprogramming, prompting CAFs to produce and secrete a broad array of pro-tumour factors, including pro-inflammatory cytokines (IL-6, IL-8), pro-fibrotic factors (TGF-β), chemokines (CXCL12), and matrix-degrading enzymes (MMP-2, MMP-9) [40,42,75–77]. Therefore, Piezo1 serves as a central hub that integrates inflammatory chemical signals with physical matrix cues, shaping an immunosuppressive, pro-fibrotic, and pro-invasive tumour microenvironment through precise regulation of CAF cytokine secretion, ultimately accelerating malignant tumour progression.

Although the Piezo1-mediated inflammation-mechanosensing axis provides a novel framework for understanding CAFs activation, its clinical translation faces significant challenges. A major limitation lies in the context dependence of its signalling pathways; Piezo1’s functional output in CAFs can vary fundamentally depending on tumour type and microenvironmental composition, raising questions about its suitability as a universal therapeutic target. Moreover, Piezo1 appears to function primarily as a signal amplifier rather than a direct driver of CAF activation. This suggests that inhibiting its activity alone may be insufficient to reverse an established fibrotic microenvironment. Furthermore, its systemic physiological expression poses a risk of on-target toxicity. Future research should therefore move beyond correlative observations and focus on defining the precise molecular thresholds that determine Piezo1 signalling outcomes. Developing strategies that selectively target its downstream effectors, rather than the channel itself, is essential to develop effective therapeutic avenues.

## 7. Piezo1 and ECM remodelling

**Figure 3.**
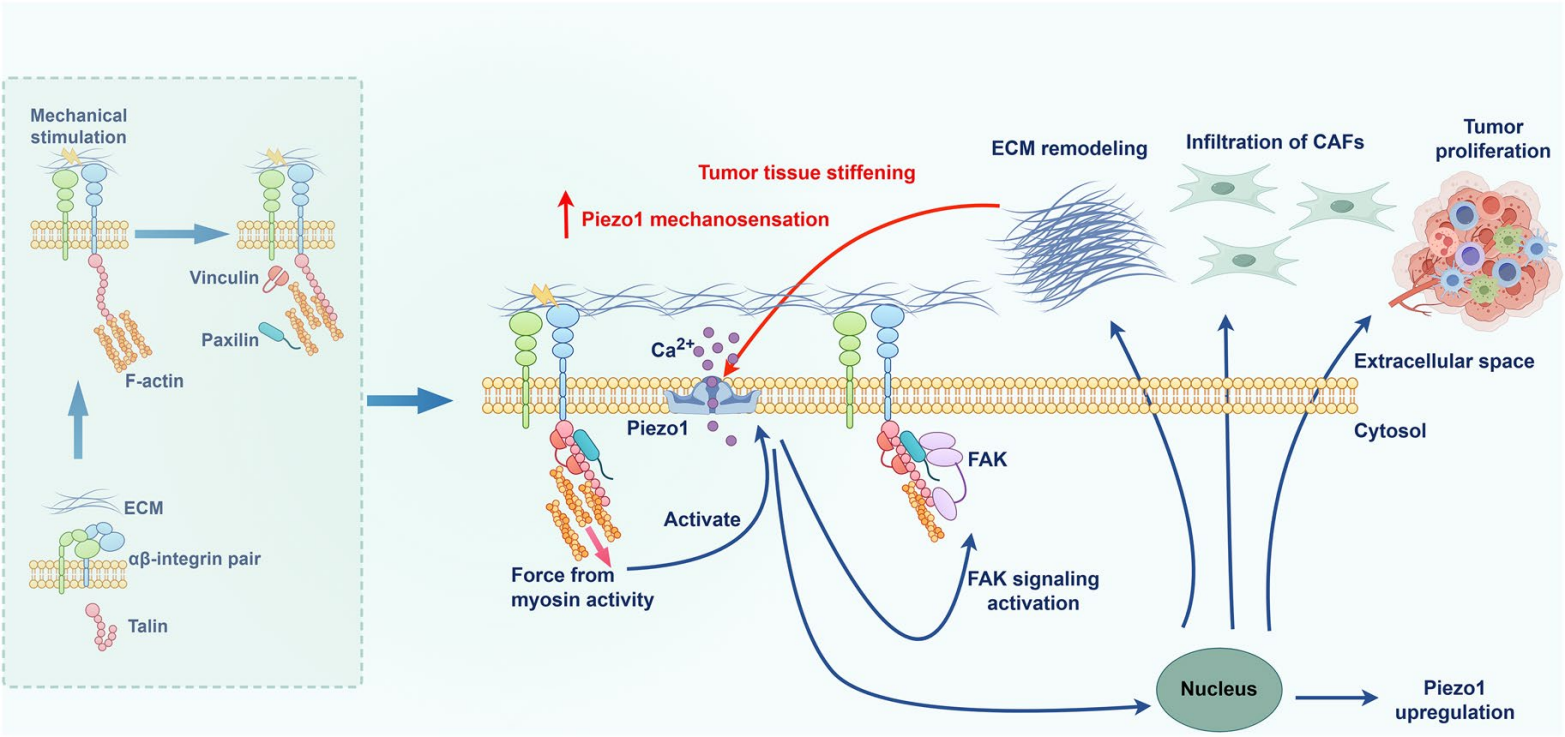
The molecular mechanism by which Piezo1 responds to mechanical stimuli and the positive feedback loop formed between it and abnormal tissue mechanics that promote tumour progression.

The ECM is a complex, dynamic structure continuously remodelled through the synthesis and degradation of its protein components and plays a crucial role in tumourigenesis [78]. During tumour development, ECM degradation releases stored growth factors and cytokines, while changes in ECM composition and abundance alter tissue density and stiffness. Both processes induce tumour cell growth, angiogenesis and inflammation [78]. Mounting evidence suggests a positive correlation between ECM stiffening-induced Piezo1 activation and cancer progression (Figure 3). In glioma [4], ECM stiffening creates a mechanical microenvironment that activates Piezo1, which in turn promotes focal adhesion assembly and stimulates the integrin-FAK signalling pathway, thereby promoting cancer cell proliferation, migration and further stiffening. This establishes a positive feedback loop between Piezo1-dependent mechanotransduction and abnormal tissue mechanics, promoting cancer progression. Consistently, mechanical stimulation activates Piezo1, thereby promoting α-SMA expression and CAF infiltration [43]. Therefore, targeting Piezo1 to modulate ECM stiffness is a potential therapeutic target in cancer treatment.

## 8. Collaboration and competition: Piezo1 within the tumour mechanosensory network

In tumour mechanobiology, the function of Piezo1 cannot be understood in isolation; rather, it operates within a complex network of mechanosensitive ion channels, including TRP channels, two-pore potassium (K2P) channels (such as TREK-1 encoded by the KCNK2 gene), and the OSCA/TMEM63 family, among others [79]. These components collaboratively decode mechanical signals in the tumour microenvironment through physical interactions and functional complementarity. Their interplay is primarily manifested at three distinct levels.

### 8.1. Physical interactions: assembly of mechanosensory complexes

Multiple mechanosensors can form functional interactomes on the plasma membrane, enabling signal cross-talk through physical proximity. For example, rapid Ca^2+^ influx and membrane depolarization triggered by Piezo1 activation can secondarily activate Ca^2+^-sensitive or voltage-sensitive TRP channels (e.g. TRPV4), amplifying and prolonging mechanical signals [80]. Simultaneously, background potassium currents through K2P channels such as TREK-1 induce membrane hyperpolarization, counteracting Piezo1-mediated depolarization. Together, these forces fine-tune cellular membrane potential, which in turn modulates the ionic driving force and activation threshold of Piezo1 [81].

### 8.2. Functional division and complementarity: decoding multidimensional mechanics

The core advantage of this mechanosensory network lies in its synergistic integration of mechanical stimuli. Piezo1 functions as a high-speed sensor, whose rapid adaptation enables precise detection of transient tensile forces and fluid shear stress, providing the cell with instantaneous information about its mechanical environment [82]. In contrast, slow-adapting channels such as TRPV4 and certain K2P channels (e.g. TREK-1) convert these transient Piezo1 signals into sustained responses to persistent matrix stiffness through their constitutive activity [83,84]. Furthermore, the OSCA family, sensitive to cell volume and osmotic pressure, operates in parallel to monitor hydrostatic pressure and osmolarity, adding another dimension to mechanical sensing [85]. This division of sensory functions and dynamic complementarity prevents Piezo1 from acting in isolation, enabling it to function within a sophisticated decoding system that integrates multidimensional mechanical cues – both dynamic and static, tensile and compressive – allowing the cell to make precise fate decisions.

### 8.3. Downstream signal integration and cell fate determination

Mechanoreceptors transmit signals primarily by regulating ion fluxes (primarily Ca^2+^ and K^+^); however, the downstream signalling networks they engage exhibit remarkable specificity and functional division. This specialization is not random but reflects each channel’s ion selectivity, subcellular localization and intrinsic protein interactome. For example, the rapid and significant Ca^2+^ influx mediated by Piezo1 efficiently activates calcium-sensitive transcriptional regulators such as NFAT and, by modulating cytoskeletal tension, significantly influences YAP/TAZ nuclear translocation. This pathway directly drives cell proliferation and invasion programs in various cancers [34,86,87]. In contrast, other channels favour distinct signalling routes. The receptor-operated TRPC6 channel, for instance, induces calcium influx and is frequently coupled with diacylglycerol signalling, establishing a preferential link to the NF-κB pathway, which primarily governs pro-inflammatory cytokine production [88]. These pathway preferences dictate cellular fate decisions: in the tumour microenvironment, mechanical signalling dominated by the Piezo1-YAP axis primarily drives gene programs associated with proliferation and migration [34,87], while co-activation of the TRPC6-NF-κB axis amplifies inflammatory responses and remodels the immune microenvironment atop the existing signals [88,89].

However, current research largely focuses on individual channels and their dominant signalling axes. A critical, yet systematically unanswered question is whether extensive cross-talk exists among these specialized pathways, potentially manifesting as synergistic, antagonistic, or hierarchical regulatory interactions. Specifically, within the mechanosensory network, does Piezo1-driven YAP/TAZ signalling directly modulate NF-κB transcriptional activity, and if so, through what mechanism? Addressing these questions is essential to clarify whether these pathways simply operate in parallel or constitute an integrated signalling network with advanced functions in complex *in vivo* microenvironments. Future research should shift from studying individual molecules to understanding the functionality of entire network modules. Targeting key nodes that regulate network balance may yield more specific and effective anti-cancer strategies than approaches focused solely on inhibiting Piezo1.

## 9. Current drugs and trials targeting Piezo1

Numerous studies have shown that Piezo1 promotes the proliferation and migration of gastric, liver, prostate and ovarian cancer cells, suggesting it as a potential therapeutic target [5,90–92]. However, Piezo1 expression varies across organs, and it response to different signals and mechanical forces is highly context-dependent, making mechanically mediated Piezo1 signalling during tumour metastasis extremely complex. This functional versatility arises not from the intrinsic complexity of the channel itself but from a multi-layered regulatory network. First, at the force-sensing level, Piezo1 is embedded within a dynamic ‘mechanobiological synapse’, where local membrane lipid composition, cholesterol content and cytoskeletal tension collectively pre-tune the channel’s sensitivity and mechanical conduction path. This enables differential responses to stimuli such as substrate stiffness, fluid shear stress, or cellular stretching [93]. Second, at the signal transduction level, these mechanical inputs are converted by Piezo1 into Ca^2+^ signals with specific spatiotemporal ‘signatures’ – including flickers, waves, or sustained plateaus – providing preliminary encoding of mechanical information [94]. Finally, at the signal interpretation and integration level, this Ca^2+^ ‘language’ is decoded by cell type-specific downstream networks. For example, in lung adenocarcinoma [95], Piezo1-Ca^2+^ signalling initiates the tumour-suppressive ROS/Wnt/β-catenin pathway, whereas in gastric cancer [43], the same signal cooperates with the YAP1/CTGF pathway to drive pro-fibrotic and inflammatory factor expression. Thus, Piezo1 functions as a universal ‘force-to-electricity’ converter, with its ultimate biological output determined by the cellular microenvironment, the type of mechanical stimulus, and the intracellular signalling networks it engages. This characteristic enables it to mediate diverse pathological processes across different stages of tumour progression. Numerous antagonists and agonists have been developed to bind Piezo1 directly or indirectly (Table 3); however, these modulators often exhibit only moderate affinity and suboptimal ‘drug-like’ properties. Consequently, research is gradually shifting towards targeting Piezo1-mediated regulation of the tumour microenvironment, particularly the tumour immune microenvironment, rather than focusing solely on tumour cells.

**Table 3.** Piezo1-related antagonists and agonists.

Drug type	Mechanism of action	Refs
**Antagonist**		
Capsaicin	Activation of the TRPV1 channel indirectly inhibits Piezos	[127]
SERCA2	Acts at the junction of intracellular ion channel structures and mechanotransduction structures to inhibit Piezo1	[128]
RR	Pore blocking occurs, inhibiting the inward MA current of Piezo1	[129]
Gd^3+^	Effectively binds intramembrane anionic phospholipids to move Piezo1 channels towards a closed state	[130]
GsMTx4	Altering the local membrane tension disrupts the efficiency of the mechanical stimulation delivered to the Piezo1 channel	[131]
Aβ peptides	Modulation of membrane structure to inhibit the response of Piezo1 channels to membrane tension and stiffness	[132]
Saturated and polyunsaturated fatty acids	Regulates Piezo1 channel protein activity and inactivates it	[133]
Dooku1	Inhibition of Piezo1-induced Ca^2+^ inward flow to reduce membrane-exposed PS levels	[134]
Tubeimoside I/Salvianolic acid B	Reversible competition with Yoda1 to bind Piezo1 and block Ca^2+^ inward flow	[135, 136]
Jatrorrhizine	Effective blockade of Piezo1-induced Ca2+ inward flow in a concentration-dependent manner	[137]
Escin	Inhibition of Yoda1-induced Ca2+ transients in endothelial cells	[138]
**Agonist**		
Yoda1	Acts on the C-terminal intracellular region of Piezo1, activating Piezo1 activity in a Ca2+-dependent manner	[139]

SERCA2, sarcoplasmic endoplasmic reticulum Ca2+ ATPaseII; RR, ruthenium red; Gd^3+^, gadolinium; GsMTx4, Grammostola mechanotoxin #4; Aβ, amyloid beta; MA, mechanically activated; PS, phosphatidylserine.

Research has shown that classical immune cells, such as MDSCs, can regulate the TME. Global inhibition of Piezo1 using peptide inhibitors reduces MDSC numbers, which can positively impact cancer progression [45]. As previously discussed, targeting Piezo1 in DCs modulates Th1 and Treg subset differentiation and function, promoting adaptive immune microenvironment remodelling and enhancing anti-tumour immunity [65].

Macrophages are the most abundant immune cells in solid tumours, can sense mechanical stress within the TME. Mechanical stimuli activate Piezo1, promoting macrophage polarization towards an immunosuppressive phenotype and inhibiting T cell tumour-killing capacity, thereby contributing to the formation of the immunosuppressive TME [96,97]. Consequently, macrophage-targeted tumour immunotherapies have garnered significant attention in recent years. However, these strategies face challenges related to tissue specificity and off-target effects. Critical questions include how to selectively target Piezo1 in specific immune cell subsets without disrupting the normal mechanosensing functions of other cells, and how to mitigate potential side effects from systemic Piezo1 inhibition or activation. Recent advances in nanocarriers and surface-modified targeting ligands may provide a means to selectively deliver Piezo1 modulators to specific cells [98]. Alternatively, developing activatable components that are released only within the TME, such as pH-, ROS-, or enzyme-sensitive Piezo1 modulators, could improve specificity [99,100]. Local delivery approaches, including intratumoural injection, implantable hydrogels, or transcatheter administration, may further reduce systematic side effects, although they are limited to localized solid tumours. Gene-editing strategies using CRISPR-Cas9 or siRNA to knock down Piezo1 in defined cell populations offer another avenue for precise therapy [101,102]. Research has shown that hyaluronidase or collagenase delivery with targeted vascular growth factors and EGFR-integrin dual-targeting drugs can interfere with ECM synthesis, specifically blocking mechanical force activation of macrophages [103–105]. In an orthotopic bladder cancer model [106], an R11 peptide-modified nanoparticle was developed: the R11 peptide forms hydrogen bonds with integrin β1, enhancing nanoparticle binding to tumour cells *via* the Piezo1/integrin β1 signalling axis. Piezo1-mediated calcium influx triggers macropinocytosis, significantly improving drug uptake. Similarly, a glutathione (GSH)-responsive mesoporous silica nanoparticle (DMON-P) loaded with pioglitazone has been used for CAF reprogramming [107]. Linking such approaches with Piezo1 modulators might enable targeting of specific TME cell subpopulations. However, these strategies have only undergone preclinical testing. Human trials are lacking, and delivery efficiency and safety remain uncertain. In conclusion, overcoming challenges of specificity and off-target effects in Piezo1-targeted therapies could represent a major breakthrough in cancer treatment.

## 10. Conclusions

As a core member of the mechanosensitive ion channel family, Piezo1-mediated mechanotransduction exerts multidimensional regulatory roles in malignant tumour progression. Recent advances in cryo-electron microscopy and structural biology have enabled preliminary resolution of Piezo1’s three-dimensional structure and mechanical gating mechanism. However, significant knowledge gaps remain regarding its dynamic allosteric regulation, finely tuned activation networks, and cross-talk with key signalling pathways within the TME. Addressing these scientific questions is critical for elucidating the profound biological significance of mechanosensing in tumourigenesis and progression.

With the paradigm shift in cancer research, the TME is increasingly recognized as a dynamic biomechanical ecosystem. This specialized niche comprises heterogeneous cell populations (including endothelial cells, immune cells, fibroblasts, etc.) and an abnormal ECM. Its unique biophysical properties, such as matrix stiffness gradients and elevated interstitial fluid pressure, are intricately coupled with biochemical signalling networks. Notably, Piezo1 decodes spatiotemporally specific mechanical stimuli within the TME, influencing malignant phenotypes such as abnormal tumour vasculature, immune evasion and metabolic reprogramming. In this review, we systematically analyzed endothelial Piezo1-mediated mechanical regulation of angiogenesis and immune cell Piezo1-driven immune checkpoint homeostasis. Based on these insights, we propose a novel ‘mechano-immuno’ synergistic targeting strategy that leverages cell type-specific Piezo1 regulatory networks. This multi-target intervention concept holds promise for overcoming current limitations in tumour therapy, where biomechanical microenvironment remodelling and immune modulation are often addressed independently.

Current Piezo1-targeted drug development faces dual challenges. First, the signalling cascade linking Piezo1 to downstream effectors (e.g. YAP/TAZ, NF-κB, HIF-1α) remains incompletely mapped. Second, existing tool compounds (e.g. GsMTx4, Yoda1) exhibit limitations in subtype selectivity and tissue targeting. Promisingly, integrating biophysical approaches, such as single-molecule force spectroscopy and super-resolution imaging, with cutting-edge platforms such as organoid-on-a-chip and spatial proteomics may facilitate the construction of quantitative models describing the ‘mechanical force-channel conformation-signal output’ relationship. This multidisciplinary strategy will not only provide a rational framework for designing allosteric modulators but also pave way for novel precision therapies targeting the tumour mechanical microenvironment.

## Data Availability

Data sharing is not applicable to this article as no data were created or analysed in this study.
